# Examining the Influence of Cultural Immersion on Willingness to Try Fruits and Vegetables among Children in Guam: The *Traditions* Pilot Study

**DOI:** 10.3390/nu12010018

**Published:** 2019-12-20

**Authors:** Tanisha F. Aflague, Rachael T. Leon Guerrero, Treena Delormier, Rachel Novotny, Lynne R. Wilkens, Carol J. Boushey

**Affiliations:** 1 College of Natural and Applied Sciences, University of Guam, Mangilao, GU 96923, USA; rachaeltlg@triton.uog.edu; 2 School of Human Nutrition, McGill University, Sainte-Anne-de-Bellevue, QC H9X 3L9, Canada; treena.delormier@mcgill.ca; 3 Human Nutrition, Food, and Animal Sciences, College of Tropical Agriculture and Human Resources, University of Hawaii at Manoa, Honolulu, HI 96822, USA; novotny@hawaii.edu; 4 University of Hawaii Cancer Center, University of Hawaii at Manoa, Honolulu, HI 96813, USA; lynne@cc.hawaii.edu (L.R.W.); cjboushey@cc.hawaii.edu (C.J.B.)

**Keywords:** child intervention, fruit and vegetable intake, Guam

## Abstract

This pilot study examined the influence of cultural immersion on willingness to try fruits and vegetables (FV) among children 3–12 years old in three summer camps in Guam with different cultural exposure levels: cultural immersion camp (CIC), high exposure; university day camp (UDC), moderate exposure; and recreational sports camp (RSC), zero exposure. Children, ages 3–12 years old for CIC and UDC and 5–12 years old for RSC, participated: CIC (*n* = 47), UDC (*n* = 23), and RSC (*n* = 33). Children’s willingness to try FV was assessed with the *Adapted WillTry* tool before and after each program. Whole FV intakes were assessed concurrently using the mobile food record in CIC and UDC. Using multivariate regression, *WillTry* post-assessment outcomes were modeled adjusting for pre-assessment, child characteristics, exposure, and parent cultural affiliation. Unique to the *Adapted WillTry* tool are three FV scales, local novel, local common, and imported, which are classified by source (local or imported) and/or familiarity (novel or common). *WillTry* adjusted mean FV post-scores by highest exposure camp to lowest were 2.2, 2.3, and 2.2 for local novel and 2.6, 2.6, and 2.6 for local common. No differences among camps were significant; however, there was an increase in the willingness to try scores for all FV score types and camps. The *Traditions* pilot study demonstrated: (1) feasibility of a multi-arm parallel design using existing community programs in limited-resource environments and (2) further examination of nutrition education components and contexts are needed to understand diet behaviors of indigenous populations.

## 1. Introduction

For the higher obesity prevalence rates in indigenous populations, including in Guam, the Chamorros compared to non-Hispanic whites correlate with chronic disease patterns [1]. Sociocultural factors, including traditional dietary habits and the cultural significance of staple foods, have the potential to influence positive health behavioral change [1,2]. Among children, common intervention strategies have been nutrition lessons, fruit and vegetable (FV) exposure or tasting, and peer modeling in school or camp settings [3,4]. Culturally adapted programs have increased children’s fruit and vegetable (FV) intake and/or preference [3,5]. One study in Guam found a culturally modified nutrition curriculum originally designed for children in the U.S. mainland was feasible and warranted [6]. 

Children’s preferences for FV was shown to be an adequate indicator of FV consumption [7]. A study among children, 3–11 years old (y), in Guam [8] adapted the previously validated *WillTry* tool that measures children’s willingness to try FV [9]. Unique to the *Adapted WillTry* tool [8] are three FV scales, local novel, local common, and imported, which are classified by source (local or imported) and/or familiarity (novel or common). Children were found to prefer imported FV more than local FV [8]. This finding parallels the nutrition transition [10,11], where diets shifted away from local traditional food systems [12], and highlights a need to examine interventions to improve preference for and intake of local (traditional) FV as a way to increase intake and variety of FV overall. 

Culture provides the context of meaning for individual behaviors and is, therefore, a critical element for influential behavioral interventions, such as FV intake, mediated by FV preference [7,13]. For many indigenous cultures, the traditional food system and cultural practices around food create opportunities for FV exposure and intake [6,12,14]. The traditional food system is food within a culture from local, natural resources [15]. Traditional diets and practices, as well as a sense of ethnic identity or pride among indigenous peoples, have been shown to protect health [15,16,17].

The *Traditions* pilot study described here examined the influence of Chamorro cultural immersion on children’s willingness to try local FV in Guam. The primary hypothesis was that children attending a cultural immersion camp with culturally adapted nutrition lessons would have a higher *Adapted WillTry* post-score than children in programs without cultural immersion. A secondary hypothesis was that these same children would also have a higher whole FV intake. This is the first study to examine whether cultural immersion influences children’s FV preference and intake in the Pacific.

## 2. Materials and Methods

A pre-post, quasi-experimental design was used to measure outcomes (i.e., willingness to try FV and FV intake) between three existing summer camp programs in Guam: cultural immersion camp (CIC), university day camp (UDC), and recreational sports camp (RSC). This pilot, multi-arm parallel design study cost-effectively used existing programs run by organizations willing to incorporate activities to answer the research question [18]. 

Children registered in the 2014 study summer camps were eligible to participate. The age range for participants was 3–12 y for CIC and UDC and 5–12 y for RSC. In RSC, only children who were registered for two, 2-week sessions were eligible. These programs each had a registration fee and were open to children of any race and ethnicity. Recruitment materials were included with camp registration packets. The Human Studies Program of the University of Hawaii (as part of the first author’s thesis effort) and the University of Guam Committee on Human Subjects approved this study.

The primary intervention was the cultural immersion within CIC. Unique to the *Traditions* pilot study were culturally adapted nutrition lessons incorporated into CIC and UDC. CIC had a high cultural dose as the *Traditions* lessons were implemented within the cultural immersion context of the camp. The UDC had moderate cultural dose from the *Traditions* lessons only. RSC was without both cultural immersion and *Traditions* lessons, or had zero cultural doses. 

The CIC and UDC programs were nearly matched for daily activities (e.g., physical activity, cooking demonstrations/taste testing, crafts) shown in Table 1. The same four *Traditions* lessons (Table 2) were incorporated into CIC and UDC and delivered by the same educators. These lessons featured local FV and promoted positive associations with eating FV. In CIC, the context of Chamorro cultural traditions, practices, and values was tied to FV. Chamorro is the language, culture, and ethnicity of Guam and the Marianas. In UDC, consuming healthy foods was introduced within a context of nutrition and human physiology. The four lessons were adapted with permission from a culturally relevant Hawaiian nutrition curriculum [19] to make them relevant to Guam and the Chamorro culture. This curriculum complemented Hawaii and Guam Department of Education K-12 Content and Performance Standards. 

The CIC activities perpetuated the Chamorro culture and language through song/chanting, dance, prayer, arts and crafts, cooking, outdoor activities, and gardening based on traditional and contemporary practices. Chamorro was spoken about 80% of the time, including the delivery of key messages in the *Traditions* lessons. All activities operated on indigenous values of respect (respetu), love (guaiya), humility (mamåhlao), reciprocity (chenchule’), and restoring harmony (inafa’maolek). The UDC activities focused on promoting healthful present-day recreational activities and global foods. The activities were an extension of the Expanded Food and Nutrition Education Program mission [20]. RSC was exclusively physical activity. Refer to Table 2. Upon completion of the last assessments, educators provided at least one *Traditions* lesson for registrants at RSC. 

Data collection occurred at two assessment periods: before (± 2 weeks) and after (± 1 week) each program. The first assessment was completed at each camp setting. The second was also completed at camp or at a pre-arranged location, such as the children’s homes or a child-friendly public space (e.g., the mall). 

*Adapted WillTry* Scores: The *Adapted WillTry* tool measures children’s willingness to try FV and was previously validated for children 3–11 years old in Guam [8,9]. The *Adapted WillTry* has 3 distinct scales of FV: local novel, local common, and imported. The scores for each scale ranged from 1 to 3, i.e., least to most willing to try. The *Adapted WillTry* administration and scoring methods have been described previously [8].

Whole FV Intake: Minimally processed FV (e.g., FV mixed dishes, fruits, vegetables) intake was assessed using the mobile food record (*mFR*) running on iOS7.1.2 on an Apple iPod touch (Apple Inc., Cupertino, CA, USA). The *mFR* is an app that has been shown to be a useful method for dietary assessment with adolescents [21,22]. This method was also tested and found to be usable for children 3–11 y in Guam [23]. Only participants in CIC and UDC were asked to use the *mFR* due to a limited number of iPods. Children used the *mFR* for two consecutive days to capture before and after images of all eating occasions. Instructions on how to use the *mFR* included taking a practice image of plastic food replicas with a fiducial marker (FM). The FM functioned as a color reference and volume marker of the food. Children were loaned the *mFR* and 2–4 FMs. Children handled the iPod, FMs, and a charging cord with a waterproof mobile carrying case. When the children returned the *mFR*, researchers asked children to assist with identifying food items that were indistinguishable, e.g., opaque containers, occluded foods; and to recall foods at eating occasions not captured as an image. 

Other Measures: Parents completed a questionnaire that included information about the child’s age, sex, language spoken, religion, and birthplace. Parent’s cultural affiliation was determined using their responses reported on a cultural affiliation questionnaire, which assesses one of four modes of acculturation: traditional, integrated, assimilated, or marginalized [24]. The same scoring system was used as described by Kaholokula and others [24]. Anthropometric assessments were completed at a time designated as least disruptive to camp activities. Height and weight were measured using a portable Seca scale and stadiometer (PE-AIM 101, Perspective Enterprises, Portage, MI, USA) using centimeters and kilograms, respectively. These measurements were converted to body mass index (BMI) as [kg / (height, m)^2^]. Dose of intervention was assessed by recording attendance at camp and at each *Traditions* lesson. 

For participation, all children were given a gift card, in $5 or $10 denominations, one before and one after camp assessments. Remuneration varied due to the different types and lengths of involvement for participants depending on the camp program.

With a sample size of *n* = 47 for CIC and *n* = 21 for UDC, with a power of 80% and a type I error rate of 0.05 (two-sided), the minimum detectable difference in means (MDD) are 0.49 for the local novel score and 0.43 for the local common score [25]. For Model 3, based on the UDC and RSC sample sizes, MDD was 0.52 and 0.49 for local novel and local common, respectively. For comparison of the secondary outcome, post-FV intake, between CIC and UDC, the MDD was 0.63, equivalent to a difference between groups of 1.3 servings per day for an SD of 1.5. 

Data were entered using a Microsoft Access (Microsoft Corporation, Redmond, WA, USA) tool specifically designed for this study. Double-data entry procedures were used and PROC COMPARE in SAS 9.3 (SAS Institute, Inc., Cary, NC, USA) was performed until both data entries achieved 100% matching. A trained analyst examined images, identified all whole FV (e.g., FV mixed dishes, fruits, vegetables), and amounts consumed. FV (100%) juices were excluded [26]. FV intake was calculated by dividing the total FV by the total number of days for which eating occasions were captured using the *mFR*.

Categorical variables were examined using frequencies and percentages and, for continuous variables, means and standard deviations were used. Doses were calculated based on *Traditions* lessons and camp attendance for CIC and UDC as the sum of days participants attended lessons and camp days divided by the total possible lesson and camp days, respectively. A dichotomous variable was created for high and low doses using the 50th percentile cut point for lessons and camp dose. 

The primary outcome was the *Adapted WillTry* FV post-scores for local novel, local common, and imported FV. The secondary outcome was post-whole FV intake assessed in CIC and UDC only. To examine the primary hypothesis, multiple linear regression models of the *Adapted WillTry* post-scores (dependent variables) were fit to examine whether and how much the *Adapted WillTry* scores differed by camp program accounting for potential confounders. A separate model was fit for each type of score: local novel, local common, and imported. Potential confounders were age (i.e., 3–6 y, 7–8 y, 9–12 y), ethnicity (i.e., Chamorro, Chamorro Mixed, Other), sex, parent’s cultural affiliation (traditional and integrated; marginalized, *n* = 2, or assimilated, *n* = 1, were eliminated due to small numbers), BMI (continuous), and attendance (i.e., high and low doses at lessons and camp). The final model excluded potential confounders that had no effect on the results and included *Adapted WillTry* pre-scores, sex, age, and ethnicity. Covariate-adjusted *Adapted WillTry* FV post-scores for each camp were computed from the models and differences between camps were evaluated with a global F-test and pairwise tests adjusted for multiple comparisons by the Tukey method. All pairwise comparisons were performed. It was hypothesized that higher cultural dose would lead to higher *Adapted WillTry* FV post-scores. In addition, *p*-values < 0.05 were considered statistically significant. 

For whole FV intake, a similar multiple linear regression model of the post-FV intake/day was used to examine differences between UDC and CIC. The final FV model was adjusted for pre-FV intake/day, age, ethnicity, and parent’s cultural affiliation. In addition, to compare with previously observed incremental trends of willingness to try FV scores [8], unadjusted difference in the pre- and post-assessment scores for each FV scale in each camp are computed from a mixed model of scores regressed on camp program, time (pre and post), and the interaction and accounting for the repeated measures. As recommended by Hochberg and Tamhane [27], all pairwise comparisons were performed. Statistical analyses were conducted using IBM SPSS Statistics version 21 (IBM Corporation, Armonk, NY, USA).

## 3. Results

**Figure 1:**
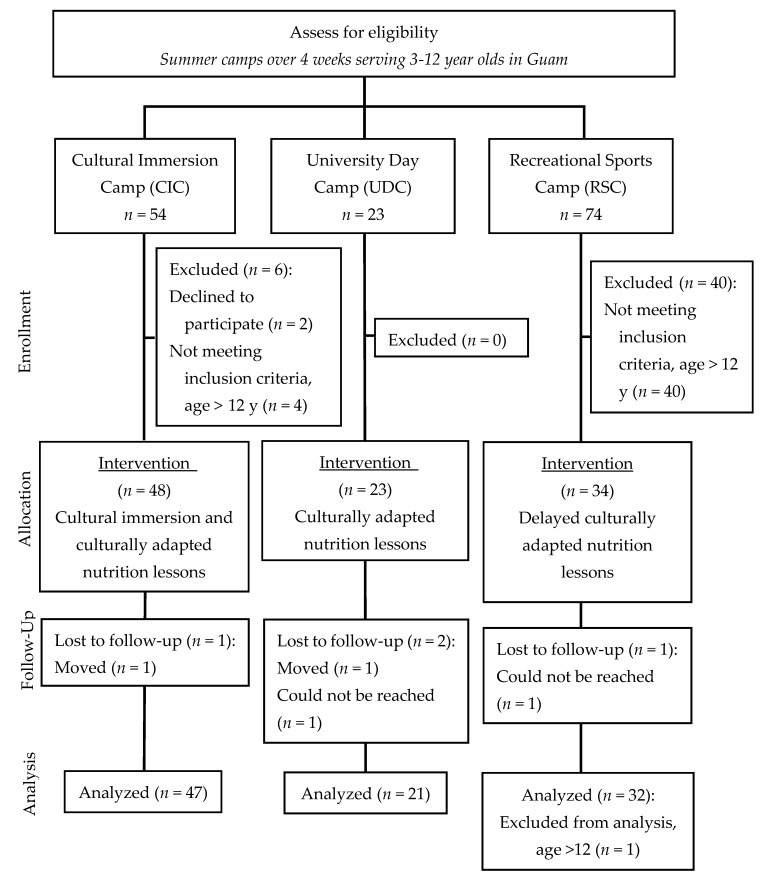
Assessment for eligibility, enrollment, allocation, follow-up, and analysis of children 3–12 years of age participating in the *Traditions* pilot study.

A total of 104 children met the eligibility criteria and agreed to participate. There were 47, 23, and 34 children from CIC, UDC, and RSC, respectively, and percent loss to follow up from the respective camps were 2%, 9%, and 3% (refer to Figure 1). Children participating in both assessment periods included 29 boys (4–11 years old) and 70 girls (3–12 years old) or 96% (99/104) of the original enrollees. The majority of the children were Chamorro as reported by parents. Refer to Table 3. For the secondary outcome of FV intake, analysis was limited to 14 boys and 43 girls that completed at least two-days of food recording using the *mFR*.

There were no significant differences at *p* < 0.05 between camp programs for the *Adapted WillTry* local novel and local common FV post-scores (refer to Table 4). The lack of significant differences among these scores remained after adjustment in multivariate analyses. The arm with the lowest cultural dose had similar post-means as the other group and even the highest score for the imported category (Table 4). 

For whole FV intake, the unadjusted means for whole FV intake/day at pre- and post-assessments were 1.2 (*n* = 23) and 0.7 (*n* = 19) for UDC and 0.6 (*n* = 47) and 0.3 (*n* = 38) for CIC, respectively. There were no significant differences in post-whole FV intakes between the camps after adjusting for the potential confounders in the final regression model (*p* = 0.86). The null relationship holds when only considering the 57 individuals with pre and post FV intakes.

For all FV score type and camps, there was an increase in the willingness to try FV scores. The exception was for UDC for Imported FV. Specifically, for Local Novel, the mean changes are 0.06 for CIC, 0.03 for UDC, and 0.04 for RSC. The corresponding changes for Local Common are 0.16, 0.11, and 0.05, and for Imported are 0.05, −0.04, and 0.06. 

## 4. Discussion

This report represents the first quasi-experiment to examine whether cultural immersion can positively influence children’s willingness to try local FV. With regard to increasing willingness to try local novel and local common FV for any of the camp comparisons, there were no statistically significant changes in either direction, i.e., decrease or increase. Interestingly, RSC had a similar or greater change in willingness to try imported FV than the other camps. Although there were no significant differences across camps, overall the *Traditions* lessons moved the FV scores higher regardless of camp, which is similar to what was observed in past studies using other methodologies. 

The change in means by camp demonstrated the consistency and robustness of the *Adapted WillTry* scores with regard to maintaining a trend (from high to low) in willingness to try imported, local common, and local novel FV that was observed in a previous study [8]. This indicates that indeed children are more willing to try imported FV than local FV and furthermore are more willing to try local FV familiar to them than other local FV that are unfamiliar to them. The *Traditions* pilot study represents the first time to use the *Adapted WillTry* tool in an intervention to capture changes in food preferences. Moreover, the lower preference for local FV underscores that interventions aimed to improve willingness to try local FV may hold promise for improving overall FV intake as children already have a high preference for imported FV. 

The *Traditions* pilot study involved community-based summer camp programs that have been in operation for, at least, seven years in Guam. This pilot study design highlights services and activities available in Guam and extends a community-based approach by enhancing local programs within their expectation and capacity. Community-based approaches are suitable for indigenous populations involving processes drawing on local knowledge to develop interventions and have been successful in the South Pacific [28]. The risk, however, is that camp programs themselves were not intended to incorporate willingness to try FV. However, the fidelity of lessons were ensured by having the same educators deliver the *Traditions* lessons. 

In general, we did not have complete control of the activities within each camp that contributed to limitations of this pilot study. All camps had predetermined schedules (e.g., duration). UDC and RSC schedules overlapped restricting FV diet assessment to only UDC due to the limited number of iPods. All camp programs had 2- or 4-week programs limiting the intervention period to 4-weeks. Using the existing community-based programs, study participants and their families had self/family-selected for the three summer camp programs. Similarly, the small sample size was due to only some of the registered camp children participating in the research activities. 

Within the CIC, the use of the Chamorro language to deliver the *Traditions* lessons may have been a limitation since CIC staff assisted with implementation and translation of lessons and nutrition concepts. Language could be a potential barrier as children likely are not fluent and are learning the Chamorro language while at camp. Nonetheless, the context of the camp in which the lessons were delivered encompassed Chamorro values and practices that may have contributed to the shift in local common FV preference. Perhaps the activities and values that were used to promote and perpetuate the Chamorro culture in the CIC do not directly involve FV, but generally all the traditional and contemporary foods of the culture. Future research involving cultural immersion should include methods and instruments that can help to capture the cultural context and impact of cultural exposure.

The traditional Chamorro food system may be transforming in turn shifting food choices parallel to the nutrition transition, creating challenges to promoting traditional local food, like FV, today. Therefore, altering children’s food preferences may extend beyond a cultural immersion program limited to the activities within the program and limited by time. However, these programs preserve traditional knowledge underpinning the value of local FV and revitalize customs that have the potential to improve local FV preference and ultimately intake. As demonstrated in this pilot study: (1) a multi-parallel design is feasible in a limited-resource environment with existing community programs, especially when examining cultural immersion that is place-based and (2) nutrition education, like *Traditions* lessons, increased willingness to try FV in all camps. Therefore, further examination of nutrition education components and educational contexts are needed to better understand willingness to try foods and diet behaviors of indigenous populations.

## 5. Conclusions

The *Traditions* pilot study demonstrated: (1) feasibility of a multi-arm parallel design using existing community programs in limited-resource environments and (2) further examination of nutrition education components and contexts are needed to understand diet behaviors of indigenous populations.

## Figures and Tables

**Table 1:** Summary of the *Traditions* pilot study camps programs philosophy, subject areas, activities, and language spoken.

Summer Day Camp Programs	Cultural Immersion Camp (CIC)	University Day Camp (UDC)	Recreational Sports Camp (RSC)
Philosophy/Mission	To promote and perpetuate the Chamorro language and culture through the implementation of immersion	Food, Fitness, & Fun: To promote physical activity and healthy foods for children and families with limited resources based on the OrganWise Guys® curricula.	To promote health, recreation, physical activity, and a lifetime of wellness in an environment with fun, cooperation, sportsmanship, and environmental awareness.
Language	Chamorro, spoken 80% of time	English	English
Core Subject Areas	Language (speaking, reading, & writing) Culture/Traditions/ValuesHistory (4 eras)	Ties healthy eating to human anatomy and physiology. Links core curricula standards: Math, language arts, and science	Daily physical activity, age-appropriate skill development in various sports, and recreational games.
Activities	Food: Cooking Chamorro food Planting/gardening of local produce using traditional and modern practices Expression of Chamorro values (e.g., respect for elders-mannginge’, restoring harmony-inafa’maolek, social reciprocity-chenchule’) Physical Activity: Dancing/Chanting Chamorro games Canoe paddling Arts & Crafts: Hut thatching/weaving Carving body ornaments Historical fieldtrips: Hiking to and camping at historical sites	Food: Cooking heart-healthy recipes Maintaining a garden to reduce cost of buying produce Foods tied to health issues and physiology Physical Activity: Contemporary activities (e.g., Zumba, bowling) Sporting games (e.g., soccer, volleyball, jump rope) Arts & Crafts: Daily food journal and related activities Fieldtrips: Store tours, outings for sports	Physical Activity: Indoor sporting and recreational activities (e.g., basketball, volleyball, dance) Outdoor sporting and recreational activities (e.g., football, softball, freeze tag) Arts & Crafts: Homemade object to use for physical activity (e.g., hacky sack/bean bag) Coloring worksheets (e.g., safe stretching) Fieldtrips Hike or outdoor excursion (e.g., waterfalls, caves, beaches) Water Park

**Table 2:** Details of the four culturally adapted nutrition lessons, *Traditions* lessons unique to this study, included local foods incorporated into the cultural immersion camp (CIC) and the university day camp (UDC) programs delivered to children aged 3–12 years old in Guam for the *Traditions* pilot study.

Lesson 1	Lesson 2	Lesson 3	Lesson 4
Food Safety & Hand Washing	A Foundation for Good Health	Label Detectives	Food Choices for Your Environment
Key Concepts: Importance of food safety Maintaining proper temperatures for Chamorro dishes Washing hands before eating	Key Concepts Protective Foods, Energy Food, Body-Building Foods, Brain Foods, Caution Foods Close to the source foods	Key Concepts Label reading Red flag food ingredients	Key Concepts Trongkon Lina’la (“Tree of Life”) Food miles Environmental impacts of food choices Environmental stewardship
Activities: 1) Handwashing song, “Biba Komplianos” (20 s) 2) Clean (Na’gasgas), Separate (Na’asangi), Cook (Fatinas), Chill (Na’manengheng) Worksheet & Activity	Activities: 1) Close to the source activity with mångga (mango) 2) Exploring the Pacific Food Guide 3) Planning a healthy breakfast	Activities: 1) Identifying Red Flag ingredients of common snack foods for children at camp 2) Measuring sugar in popular beverages in Guam	Activities: 1) Comparing food miles between local banana (chotda/aga’ håya) and banana from US Mainland (aga’ lagu) 2) Evaluate ways to keep the environment safe
Recipe: Breadfruit kabobs: steamed breadfruit dipped in warm coconut milk	Recipe: Eggplant (egg) scramble	Recipe: Papaya and/or mango smoothie	Recipe: Soursop popsicles and frozen or dehydrated star fruit

**Table 3:** Characteristics of children in the *Traditions* pilot study in Guam by camp program, ages 3–12 years old that completed the *Adapted WillTry* at pre and post (*n* = 99).

Child Characteristics	*n*	CIC ^1^ (*n* = 46)	UDC ^2^ (*n* = 21)	RSC ^3^ (*n* = 32)
Sex		*n* (percent, %)		
Boys	29	17 (37)	1 (5)	11 (34)
Girls	70	29 (63)	20 (95)	21 (66)
Ethnic Group				
Chamorro, only	53	25 (54)	12 (57)	16 (50)
Chamorro, mixed	33	21(46)	3 (14)	9 (28)
Other	12	0 (0)	6 (29)	6 (19)
No response	1	0 (0)	0 (0)	1 (3)
Age Group, years				
3–6	28	17 (37)	1 (5)	10 (31)
7–8	39	14 (30)	8 (38)	17 (53)
9– 12	32	15 (33)	12 (57)	5 (16)
Weight status, BMI ^†^ percentile				
Underweight, <5th percentile	3	2 (4)	1 (5)	0 (0)
Healthy weight, 5 to <85th percentile	66	28 (61)	16 (76)	22 (69)
Overweight, ≥85 to <95th percentile	16	9 (20)	3 (14)	4 (12)
Obese, ≥95th percentile	14	7 (15)	1 (5)	6 (19)
Parent’s cultural affiliation				
Traditional	15	6 (13)	5 (23)	4 (12)
Integrated	79	38 (83)	14 (67)	27 (84)
Marginalized	2	2 (4)	0 (0)	0 (0)
Assimilated	1	0 (0)	1 (5)	0 (0)
No response	2	0 (0)	1 (5)	1 (3)
Pre-Assessments		Mean ± SD	Mean ± SD	Mean ± SD
*Adapted WillTry* (unadjusted) scores				
Local Novel		2.2 ± 0.6	2.4 ± 0.4	2.2 ± 0.6
Local Common		2.4 ± 0.6	2.5 ± 0.4	2.6 ± 0.4
Imported		2.6 ± 0.4	2.9 ± 0.2	2.8 ± 0.3
Whole FV ^‡^ (servings)		0.6 ± 0.5 ^a^	1.2 ± 1.1 ^b^	n/a ^#^

^1^ CIC, Cultural Immersion Camp (high dose); ^2^ UDC, University Day Camp (moderate dose); ^3^ RSC, Recreational Sports Camp (zero dose). ^a^ *n* = 47; ^b^ *n* = 23. ^†^ Body Mass Index; ^‡^ fruits and vegetables; ^#^ not applicable.

**Table 4:** Adjusted post means for *Adapted WillTry* score and fruit and vegetable intake among children, 3–12 years old, in Guam by *Traditions* pilot study camp program.

	Post Means ^1^				*p*-Value for Pairwise Comparisons ^3^		
	CIC (*n* = 47)	UDC (*n* = 21)	RSC (*n* = 32)	Global *p*-value ^2^	CIC vs. UDC	CIC vs. RSC	USC vs. RSC
Local Novel *Adapted WillTry* score	2.2	2.3	2.2	0.87	0.89	1.00	0.87
Local Common *Adapted WillTry* Score	2.6	2.6	2.6	0.97	1.00	0.96	0.99
Imported *Adapted WillTry* Score	2.7	2.6	2.8	0.06	0.56	0.27	0.06
Whole FV (servings)	0.3 ^a^	0.7 ^b^	n/a ^‡^	0.25	0.25	n/a	n/a

^1^ Adjusted by regression for age, sex, ethnicity, and pre-assessment scores. ^2^ Based on F-test comparing all three camps, except for Whole FV (i.e., CIC and UDC) ^3^ Adjusted for multiple comparisons by the Tukey method. ^a^ *n* = 38; ^b^ *n* = 19. ^‡^ not applicable.
